# Neural Wiskott-Aldrich syndrome protein (nWASP) is implicated in human lung cancer invasion

**DOI:** 10.1186/s12885-017-3219-3

**Published:** 2017-03-28

**Authors:** Bethan A. Frugtniet, Tracey A. Martin, Lijian Zhang, Wen G. Jiang

**Affiliations:** 1 Cardiff China Medical Research Collaborative, School of Medicine, Cardiff University, Cardiff, CF14 4XN UK; 2 Department of Thoracic Surgery, Peking University Cancer Hospital and Beijing Cancer Institute, Key Laboratories of Carcinogenesis and Translational Research (Ministry of Education), Beijing, China

**Keywords:** nWASP, Lung, Cancer, Invasion, Wiskostatin

## Abstract

**Abstract:**

Lung cancer is one of the most commonly diagnosed cancers with survival much lower in patients diagnosed with distal metastases. It is therefore imperative to identify pathways involved in lung cancer invasion and metastasis and to consider the therapeutic potential of agents that can interfere with these molecular pathways. This study examines nWASP expression in human lung cancer tissues and explores the effect of nWASP inhibition and knockdown on lung cancer cell behaviour.

**Methods:**

QPCR has been used to measure nWASP transcript expression in human lung cancer tissues. The effect of wiskostatin, an nWASP inhibitor, on A-549 and SK-MES-1 lung carcinoma cell growth, adhesion, migration and invasion was also examined using several in vitro functional assays, including ECIS, and immunofluorescence staining. The effect of nWASP knockdown using siRNA on particular behaviours of lung cancer cells was also examined.

**Results:**

Patients with high levels of nWASP expression in tumour tissues have significantly lower survival rates. nWASP transcript levels were found to correlate with lymph node involvement (*p* = 0.042). nWASP inhibition and knockdown was shown to significantly impair lung cancer cell growth. nWASP inhibition also affected other cell behaviours, in SK-MES-1 invasion and A-549 cell motility, adhesion and migration. Paxillin and FAK activity are reduced in lung cancer cell lines following wiskostatin and nWASP knockdown as shown by immunofluorescence and western blot.

**Conclusions:**

These findings highlight nWASP as an important potential therapeutic target in lung cancer invasion and demonstrate that inhibiting nWASP activity using the inhibitor wiskostatin can significantly alter cell behaviour in vitro.

## Background

Lung cancer is one of the most commonly diagnosed cancers accounting for 13% of total cases worldwide [1]. It is also one of the leading causes of cancer death globally with survival rates much lower in patients diagnosed with distal metastases [2, 3]. This highlights the importance of understanding the mechanisms involved in lung cancer metastasis and considering how molecular pathways involved in this process could form novel potential therapeutic targets.

The critical initial steps in lung cancer metastasis involves the detachment and invasion into the surrounding tissues of tumour cells which requires changes to their adhesive and migratory properties [4]. This is achieved partly through cell polarisation and the extension of actin-rich membrane structures in the direction of movement such as filopodia, lamellipodia or invadopodia which are found in invasive cancer cells. Focal adhesions on the leading edge of these protrusions connect the actin cytoskeleton in the migrating cells to their surroundings through the coordination of numerous signalling and structural proteins, such as integrins, focal-adhesion kinase (FAK) and paxillin, allowing them to gain traction and move [5]. The formation of membrane protrusions, which are crucial for cell motility, is controlled by the rearrangement of the actin cytoskeleton [6–10].

nWASP is a 65kDa cytoplasmic protein which responds to several cellular signalling molecules to mediate actin polymerisation through interactions with the Actin-related protein 2/3 (Arp2/3) complex. When inactive, nWASP exists in an auto-inhibited, folded confirmation whereby the main catalytic domain, the VCA domain on the C-terminus, is shielded by the N-terminus regions. Signalling molecules, such as the small GTPase Cdc42, bind to and activate nWASP by destabilising the auto-inhibited state and exposing the VCA region allowing interactions with the Arp2/3 complex which, when bound to nWASP in conjunction with an actin monomer, becomes activated and actin polymerisation can be initiated [11–15]. Through this role, as a reorganiser of the actin cytoskeleton, nWASP has been implicated in the control of many cellular processes such as vesicle trafficking, pathogen infection and neurite extension to name a few. However, more interestingly with respect to cancer studies, nWASP has been shown to be involved in changes to cell morphology, such as invadopodium formation, growth and also correlates with certain cancer phenotypes. Hence, nWASP has been highlighted as a potential therapeutic target in a range of contexts, particularly in the control of cancer progression [11, 15–23].

The primary aim of this investigation is to explore the role and therapeutic potential of targeting nWASP with reference to lung cancer. This is achieved by examining the activity of nWASP in human lung cancer tissues and by studying the effects of nWASP knockdown and the nWASP inhibitor wiskostatin [24] on lung cancer cell behaviour, with particular focus towards migratory, invasive, adhesive and proliferative properties.

## Methods

### Cell lines, culture conditions and tissue samples

A-549 and SK-MES-1 lung carcinoma cell lines were purchased from ATCC (VA, USA) in October 2014 and were cultured in Dulbecco’s Modified Eagle Medium (DMEM) (Sigma-Aldrich, Dorset, UK) supplemented with 10% foetal bovine serum (FBS). Authentication of both cell lines took place using STR profiling techniques and regular testing for mycoplasma contamination was also carried out.

### Ethics, consent and permissions

Fresh frozen lung carcinoma tissues at TNM stages of 1 to 3, with matched normal tissues, were obtained from 150 patients who received curative resection in Peking University Cancer Hospital from January 2001 to December 2006. Ethical approval was provided by hospital’s Ethics Committee at Peking University Cancer hospital. Tissues were collected immediately after surgical resection and stored in the Tissue Bank of Peking University Oncology School. Clinicopathological factors, including age, sex, histological types of tumours, TNM stage, lymph node metastasis and survival were recorded and stored in the patients’ database.

### siRNA transfection

Cells were seeded in a 24-well plate in serum-free DMEM (no antibiotics) at 2 × 10^5^ cells/well. After 24 h cells were transfected with 0.17 μg nWASP siRNA (sc36006, Santa Cruz Biotechnology Inc., USA), or non-targeting siRNA (NT), delivered in antibiotic free DMEM supplemented with 5% FBS with 1 μl Lipofectamine 3000 reagent (ThermoFisher Scientific, MA, USA) per well. After a further 24 h cells were then used for RNA/protein extraction or functional assays.

### RNA isolation and QPCR

Total RNA was isolated from the homogenized tissues (150 pairs of specimens) or from cultured cells using Total RNA Isolation Reagent (ABgene™). Reverse transcription was performed using the Reverse Transcription kit (Primer design). QPCR was performed on the Icycler IQ5 system (Bio-Rad, Hammel Hemstead, UK) to quantify the level of nWASP transcripts in the samples (shown as copies/μl from internal standard normalised to actin). The QPCR technique utilised the Amplifluor system™ (Intergen Inc., England) and QPCR master mix (BioRad). nWASP QPCR primers: Forward: 5’AGTCCCTCTTCACTTTCCTC’3 and Reverse: 5’ACTGAACCTGACCGTACAACATCTCTGTGGATTGTCCT’3. Real-time QPCR conditions were 95 °C for 15 min, followed by 60 cycles of 95 °C for 20 s, 55 °C for 30 s and 72 °C for 20 s. nWASP transcript expression was then analysed and correlated with patient’s pathological and clinical information.

### PCR and Gel Electrophoresis

PCR was carried out using the following cycling conditions: 94 °C for 5 min, then 32 cycles of 94 °C for 30s, 55 °C for 40s, 72 °C for 60s with a final extension of 10 min at 72 °C. nWASP primers used: Forward: 5’AGTCCCTCTTCACTTTCCTC’3 and Reverse: 5’GCTTTTCCCTTCTTCTTTTC’3. GAPDH primers: Forward: 5’GGCTGCTTTTAACTCTGGTA’3 and Reverse: 5’GACTGTGGTCATGAGTCCTT’3. The products were run on a 2% agarose gel and visualised using SYBR safe (Abnova, Taiwan).

### Reagents and treatments

Wiskostatin (Enzo Life Sciences, Exeter, UK) was dissolved in 30% dimethyl sulfoxide (DMSO, Sigma-Aldrich) in normal cell culture medium to a stock concentration of 300 μM. Control reactions received appropriate DMSO treatments. For immunofluorescence and western blot assays, the following primary antibodies were used: paxillin BD610052 (BD Biosciences, Oxford, UK), FAK BD610058 (BD Biosciences, Oxford, UK), pFAK sc11766 (Santa Cruz Biotechnology Inc., USA), nWASP NBP1-82512 (Novus Biologicals, Abingdon, UK) and GAPDH sc32233 (Santa Cruz Biotechnology Inc., USA). The secondary antibodies, AlexaFluor 594 and AlexaFluor 488 (donkey IgG; Life Technologies, Paisley, UK) were used to conjugate to primary antibodies in immunofluorescence assays. DAPI (Merck Millipore, Watford, UK) was used to visualise nuclei. Anti-goat/−mouse/−rabbit IGG whole molecule peroxidase antibodies (Sigma Aldrich) were used to conjugate to primary antibodies in Western blot assays.

### Protein extraction, SDS-PAGE and Western Blot

Lysis buffer was used to extract protein from cells which was then used for SDS-PAGE. Proteins were transferred onto Immobilon® PVDF membranes (Merck Millipore, Watford, UK) which were blocked and probed with primary antibodies and then incubated with the corresponding peroxidase conjugated secondary antibodies (1:1000). Proteins were visualised using EZ-ECL Kit (Biological Industries, Israel).

### In vitro growth assay

Cells were seeded into a 96-well plate with appropriate treatments at a density of 3000 cells in each well with 10 replicates per treatment. After 1, 2, and 3 day incubation periods, cells were fixed using 4% formalin (Sigma-Aldrich) and stained with 1% crystal violet (Sigma-Aldrich). After washing, crystal violet was extracted from cells using 10% acetic acid (Sigma-Aldrich) in distilled water (*v*/v). Absorbance was determined at 540 nm wavelength on an absorbance plate reader (Biotek ELx800).

### In vitro cell adhesion assay

Wells on 96-well plates were pre-coated with Matrigel basement membrane matrix (BD Biosciences) at 50 μg/ml in normal culture medium. Following rehydration, 8 × 10^5^ cells, which had been incubated overnight in treatments, were then seeded into each well onto the Matrigel membrane in 200 μl of normal medium containing treatments with at least 6 replicates per sample and incubated for 25 min. Adherent cells fixed and stained as described above and visualised under the microscope at ×5 magnification.

### In vitro scratch assay

7 × 10^5^ cells were seeded in appropriate treatments into each well on a 24-well plate with at least 3 replicates per experiment. Upon reaching confluence the monolayer was scratched to create a linear wound. The plate was placed in an EVOS® FL Auto Imaging System (Life Technologies, Paisley, UK) which maintained the plate in normal culture conditions throughout the experiment. Images were captured of the wound every 30 min for up to 24 h.

### ECIS assay

Z-theta models of the ECIS (electric cell-substrate impedance sensing) instruments (Applied Biophysics Inc., NJ, USA) were used to electrically monitor coverage of gold electrodes on the base of a 96W1E+ arrays by measuring the capacitance at 64 kHz. The plate was stabilised and 8 × 10^4^ cells/well were seeded in treatments where appropriate. At least 4 replicate well were used for each sample in every experiment. An electrical wound was applied after 35 h with settings: 20s, current of 2400 μA and frequency of 60,000 Hz.

### Cytodex-2 bead motility assay

Cells were incubated at a density of 7 × 10^5^ cells/ml in normal culture medium, containing 100 μl of cytodex-2 beads (Sigma-Aldrich at 20 mg/ml in BSS), for 4 h to allow the cells to adhere to the beads. Following washes, 100 μl of the cell/bead suspension was added to a 96-well plate and incubated for 18 h in treatments with 6 replicates. Cells which had migrated from the beads to the plate were fixed, stained and counted according to absorbance as above.

### In vitro invasion assay

Cell culture inserts (8 μm pore ThinCert™ 24-well plate inserts, Greiner Bio-One GmbH, Austria) were placed into a 24-well plate and coated with Matrigel basement membrane matrix (BD Biosciences) at 50 μg/ml in normal culture medium. Cells were seeded into inserts at a density of 3 × 10^4^ cells per insert in 200 μl containing treatments with 3 replicates. After 3 days cells which had invaded through the Matrigel and migrated through the pores on the inserts were fixed, stained and counted according to absorbance as above.

### Immunofluorescence staining

For immunofluorescence staining, cells were cultured in Millicell EZ 8-well chamber slides (Merck Millipore, Watford, UK) for 18 h at a seeding density of 5 × 10^4^ cells per well. Cells were fixed in 500 μl of ice cold 100% ethanol at −20 °C. Cells were permeabilised with 0.1% Triton X 100 (Sigma Aldrich). Primary antibodies (diluted to 1:100) and secondary antibodies (1:500 for Alexa secondary antibodies and 1:1000 for DAPI) were prepared in blocking buffer in blocking buffer and applied. Slides were washed and mounted using FluorSave (Calbiochem, Nottingham, UK) and visualised using an Olympus BX51 microscope with a Hamamatsu Orca ER digital camera at × 40. Integrated density was measured using ImageJ.

### Statistical analysis

Statistical analysis of patient qPCR data was performed using SPSS software (SPPS Inc.). The relationship between nWASP and patient clinicopathological information was assessed using student’s unpaired t-tests. Multivariate analysis was carried out using Minitab. Survival curves were produced and analysed using the Kaplan-Meier method and Wilcoxon (Gehan) statistics. Other data are presented as mean ± SD. Each experiment was conducted at least 3 times and representative data are presented. Unpaired t-tests and 2-way ANOVA tests were used to statistically analyse other experimental data. A *p*-value <0.05 over at least 3 independent repeats was considered statistically significant.

## Results

### nWASP expression correlates with patient survival

**Fig. 1:**
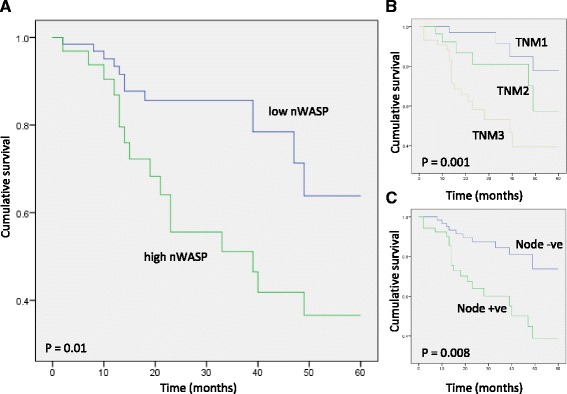
Survival of lung cancer patients according to clinicopathological parameters. **a** Survival is significantly lower in patients that exhibit high nWASP expression in tumours (*p* = 0.01). **b** Survival correlates with TNM staging significantly (*p* = 0.001). **c** Survival is significantly lower in patients which exhibit nodal activity compared with node negative patient tumour samples (*p* = 0.008)

Multivariate analysis and Kaplan-Meier survival curve analysis of various lung cancer clinicopathological groups versus survival demonstrated that TNM staging and nodal status significantly correlates with patient survival, as expected, Table 1, Fig. 1b, c. nWASP transcript levels in tumour samples are also shown to be a factor that correlates with patient survival, with survival significantly lower in patients which exhibit high levels of nWASP expression, Table 1, Fig. 1a.

**Table 1:** Multivariate analysis of lung cancer categories against overall survival

Variables	F value	*P* value
Tumour type	0.293	0.590
Differentiation	0.481	0.490
Nodal status	7.778	*0.007*
TNM staging	7.737	*0.007*
nWASP	9.546	*0.003*

Multivariate analysis was carried out to examine clinicopathological parameters against overall survival of the patients. TNM staging was found to be significantly linked to patient survival as is expected (*p* = 0.007). Likewise, nodal involvement was found to be related to overall survival (*p* = 0.007). nWASP expression is also significantly linked to patient survival (*p* = 0.003)

Significant values are shown in italics

### nWASP expression correlates with lung cancer clinicopathology

**Fig. 2:**
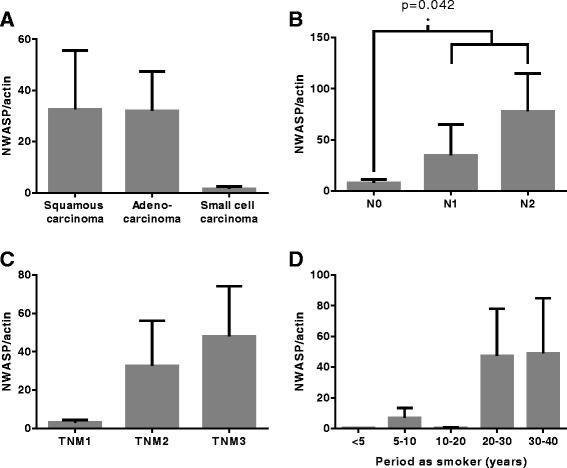
nWASP expression in lung tumour clinical samples. nWASP transcript levels normalised to actin according to clinicopathological information. **a** nWASP transcript levels are higher in both squamous and adenocarcinoma tumours compared with small-cell carcinoma samples (*p* = 0.18 and *p* = 0.052 respectively). **b** nWASP is expressed at significantly higher levels in tumour tissues which exhibit nodal activity compared with lymph node-negative tumours (*p* = 0.042). **c** nWASP transcript expression increases according to tumour grade, *p* = 0.094 for TNM1 vs. TNM3. **d** nWASP expression is higher in lung tumours where patients have a long-term smoking habit. Mean ± SEM are shown

Higher levels of nWASP transcript were detected in squamous carcinoma and adenocarcinoma tumour tissues compared with small-cell carcinoma tumour samples, Fig. 2a. nWASP also correlated with lymph node involvement and TNM stage with node positive samples having significantly higher levels of nWASP expression than node negative samples, Table 2 and Fig. 2b, c.

**Table 2:** Levels of nWASP transcripts in lung tumour tissues

Category		*N*	nWASP level (mean)	SEM
Tumour type	Squamous carcinoma	51	32.6	23
	Adenocarcinoma	68	32.1	15.3
	Small-cell carcinoma	3	1.615	0.886
Differentiation	High	7	42.8	42.6
	Medium High	16	18.84	9.69
	Medium	50	45.8	25.1
	Medium/low	23	35.5	30.1
	Low	16	5.13	3.78
Nodal status	Negative	70	8.37	2.9
	Positive	62	62.3	25.7
TNM staging	stage-1	39	3.12	1.33
	stage-2	31	32.8	23.4
	stage-3	52	48	26.2
Period as smoker	<5 years	2	0.57724	0.0007
	5-10 years	6	7	6.39
	10-20 years	19	0.521	0.217
	20-30 years	29	47.4	30.6
	30-40 years	21	49.0	35.9

Mean nWASP transcript expression normalised to actin, SEM and number of patients in category is shown. Expression is presented according to tumour type, level of differentiation, nodal involvement, tumour stage and patient smoking habit

### nWASP expression in lung tumour tissues and relationship with patient smoking habits

nWASP expression appears to correlate with patient smoking habits with long-term smokers that have a smoking habit of 20-40 years showing increased nWASP levels compared with patients who have smoked for less than 20 years, Table 2, Fig. 2d.

### Knockdown of nWASP in A-549 and SK-MES-1 cell lines

**Fig. 3:**
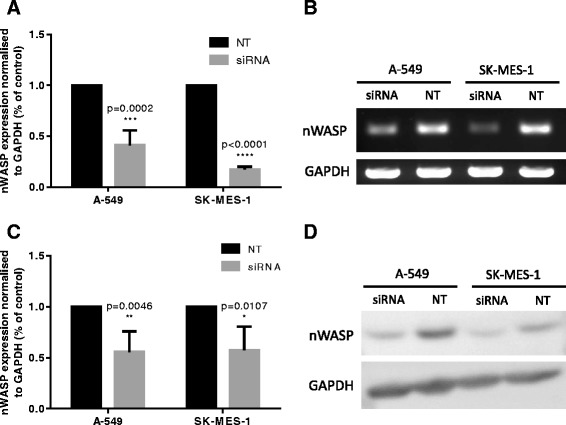
Generation of nWASP knockdown cell lines. A-549 and SK-MES-1 cells were treated with nWASP siRNA/non-targeting siRNA (NT) at 0.5 μg/ml and then analysed for nWASP expression after 48 h. **a** QPCR analysis of nWASP transcript expression demonstrates a significant decrease in expression in siRNA treated cells, *n* = 4 replicates. **b** PCR also demonstrates a decrease in nWASP expression. **c** Quantitative analysis of Western blots (*n* = 4) shows significant decrease in nWASP protein expression in both A-549 and SK-MES1 cell lines after 48 h siRNA treatment. **d** Representative image showing knockdown of nWASP at protein level in siRNA treated cells at 48 h

Since nWASP transcript expression is elevated in adenocarcinoma and squamous carcinoma cell lines (Fig. 2a), A-549 and SK-MES-1 cell lines were selected as models of these lung cancer sub-types respectively in order to examine the role of nWASP. nWASP siRNA was used to significantly knockdown nWASP at 48 h at both gene and protein level as demonstrated by qPCR, PCR (Fig. 3a, b) and Western blot (Fig. 3c, d).

### ECIS analysis of nWASP inhibition and knockdown in A-549 and SK-MES-1 cells

**Fig. 4:**
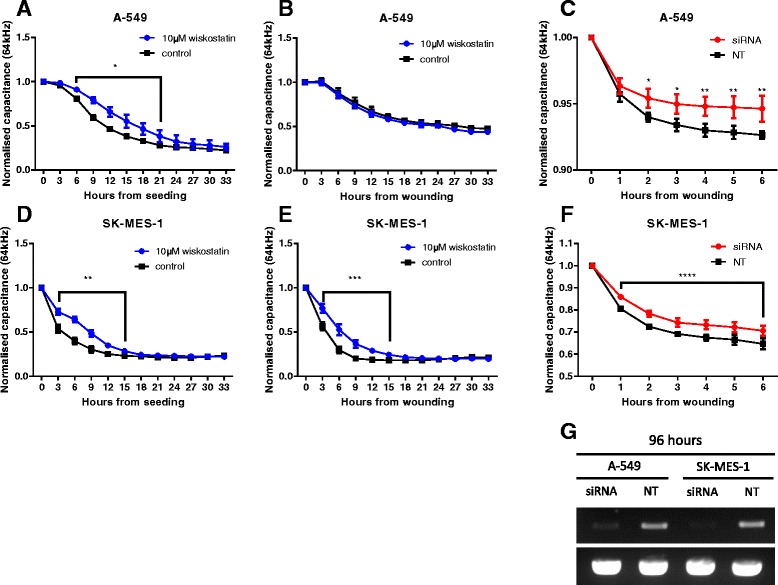
The effect of wiskostatin and nWASP knockdown on cell behaviour monitored using ECIS. **a** Wiskostatin treatment significantly increases the capacitance of A-549 cells following seeding. **b** No significant effect is detected following wounding in A-549 cells. **c** nWASP knockdown causes a significant increase in capacitance following an electrical wounding. **d** Wiskostatin also causes a significant increase in capacitance following seeding in SK-MES-1 cells. **e** A significant increase in capacitance is also detected in SK-MES-1 cells following an electrical wounding in wiskostatin treated cells. **f** This increase in capacitance is also shown in SK-MES-1 nWASP knockdown cells. **g** PCR using A-549 and SK-MES-1 nWASP knockdown cells following ECIS demonstrates that decreased nWASP expression in siRNA treated cells is still present at 96 h compared with cells treated with the non-targeting control siRNA. Significant differences between samples are represented by * (*P* < 0.05), etc. over the appropriate time-points

ECIS analysis of A-549 and SK-MES-1 cell lines following wiskostatin treatment showed that the capacitance following seeding was significantly increased compared with the controls in both cell lines, Fig. 4a, d respectively. This suggests that the initial attachment and spreading behaviour of these cells is impaired by nWASP inhibition. Following an electrical wounding, SK-MES-1 cells are found to have a significantly increased capacitance following nWASP inhibitor treatment, Fig. 4e. No significant difference between the capacitance of the wiskostatin treated and control A-549 cells is detected, Fig. 4b. This suggests that the invasive/migratory capacity of SK-MES-1 cells following an electrical wounding is impaired by nWASP inhibition but not in A-549 cells. ECIS analysis of the healing of an electrical wound using A-549 and SK-MES-1 nWASP knockdown cell lines demonstrates that capacitance is significantly increased in both cell lines following siRNA treatment, Fig. 4c, f respectively. Figure 4g shows that nWASP knockdown is still present in both cell lines 96 h after siRNA treatment, the time period over which the ECIS assay takes place.

### nWASP affects in vitro growth of lung cancer cells

**Fig. 5:**
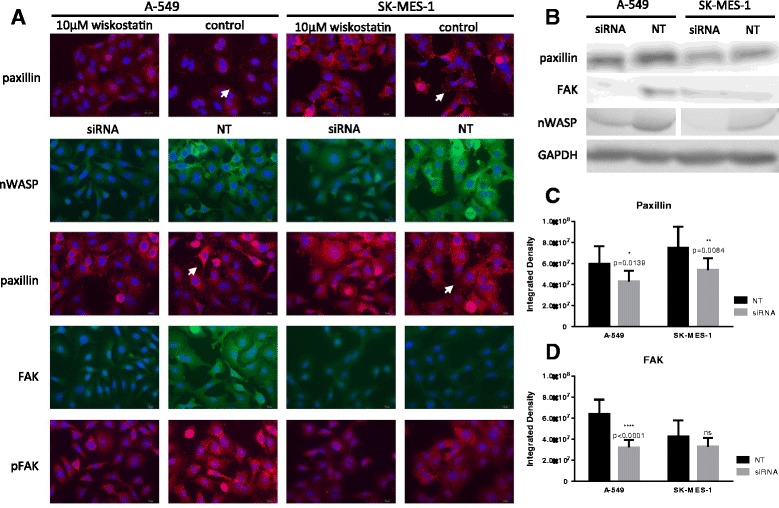
nWASP activity affects A-549 and SK-MES-1 cell growth. **a**, **b** nWASP inhibition using 10 μM wiskostatin treatment significantly impairs the growth of A-549 and SK-MES-1 cells, respectively. **c**, **d** A significant effect on growth is also evident after 3 days in nWASP knockdown A-549 and SK-MES-1 cells, respectively, when compared to non-targeting control treated cells

10 μM wiskostatin treatment was seen to significantly inhibit cell growth over 3 days in vitro in both A-549 and SK-MES-1 cells according to the absorbance, Fig. 5a, b respectively. This effect on growth was also shown in nWASP knockdown A-549 and SK-MES-1 cell lines, with a significant difference detected after 3 days, Fig. 5c, d respectively.

### In vitro motility, migration and adhesion of A-549 cells are reduced following wiskostatin treatment

**Fig. 6:**
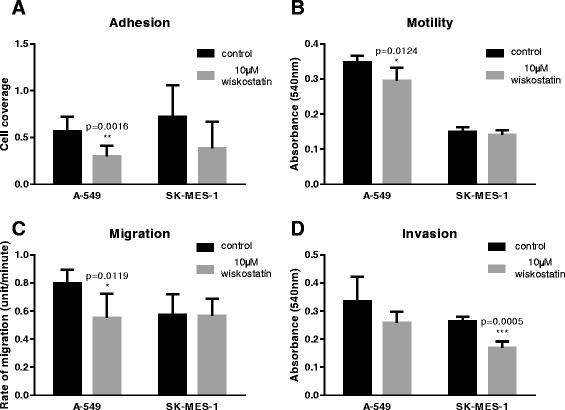
In vitro effect of wiskostatin on A-549 and SK-MES-1 invasion, adhesion, motility and migration. **a** Wiskostatin treatment impairs the ability of A-549 and SK-MES-1 to adhere to a Matrigel basement membrane over a 25 min period although only with significance in the former cell line. **b** From cytodex-2 motility assays, A-549 cell motility found to be significantly impaired by wiskostatin treatment but SK-MES-1 motility is not significantly affected. **c** The rate of closure of a scratch wound is reduced in A-549 cells following wiskostatin treatment but no significant effect on the rate of migration is seen in SK-MES-1 cells. **d** The capacity of A-549 and SK-MES-1 cells to invade through pores on a Matrigel lined insert is impaired by wiskostatin treatment but only significantly in SK-MES-1 cells

Motility and migration in A-549 cells was seen to be significantly reduced following 10 μM wiskostatin treatment according to the number of motile cells measured and the rate of scratch wound closure, Fig. 6b, c respectively. Although motility was consistently reduced, no significant difference in motility or migration was found between wiskostatin treated and control SK-MES-1 cells, Fig. 6b, c. The level of adhesion was consistently reduced in both cell types when treated with wiskostatin; however this finding was only consistently significant in A-549 cells, Fig. 6a.

### In vitro invasion of SK-MES-1 cells is affected by wiskostatin treatment

The invasive capacity of A-549 and SK-MES-1 cells was reduced following wiskostatin treatment according to the in vitro invasion assay data, Fig. 6d, but only significantly over all repeats with SK-MES-1 cells. ECIS experiments support this finding where the capacitance is increased significantly in SK-MES-1 nWASP knockdown and wiskostatin treated cells, Fig. 4e, f.

### Focal adhesions are affected by nWASP activity in lung cancer cells

**Fig. 7:**
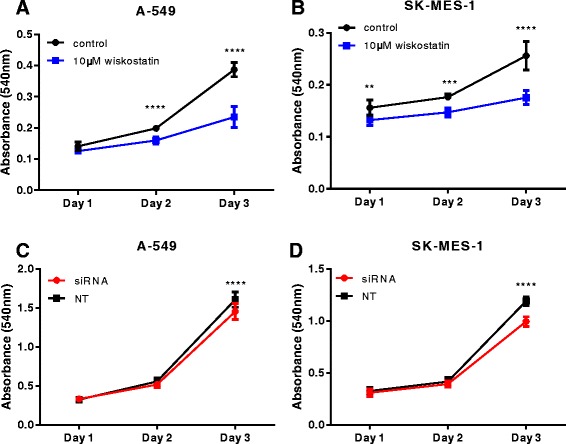
Protein changes in response to nWASP inhibition and knockdown. **a** Immunofluorescence staining for paxillin, FAK, pFAK and nWASP in response to nWASP knockdown and the effect on paxillin in wiskostatin treated A-549 and SK-MES-1 cells. Arrows point to paxillin rich adhesions (**b**) Western blot demonstrating the effect on paxillin and FAK in response to nWASP knockdown in A-549 and SK-MES-1 cell lines. **c**, **d** Quantitative analysis of immunofluorescence staining in A-549 and SK-MES-1 nWASP knockdown cells for paxillin and FAK respectively, (mean + SD, *n* = 10 replicates)

Paxillin activity was considerably altered following wiskostatin treatment and nWASP knockdown with fewer paxillin rich adhesions on the perimeter of the treated cells compared with the controls, Fig. 7a. A decrease in paxillin activity which correlates with nWASP activity has also been shown through Western blot analysis of A-549 and SK-MES-1 knockdown cells after 48 h of siRNA treatment, Fig. 7b. Paxillin expression was quantified by measuring the integrated density and found to be significantly reduced in A-549 and SK-MES-1 siRNA treated cells compared with the non-targeting control treated cells, Fig. 7d. In addition, FAK activity was examined in response to nWASP knockdown. FAK and pFAK was reduced in A-549 nWASP siRNA treated cells compared with the control cell line, Fig. 7a, b, d. FAK and pFAK (Tyr 925) levels seemed lower in SK-MES-1 cells and were not significantly affected by nWASP activity, Fig. 7a, b, d.

## Discussion

Analysis of nWASP transcript expression highlights several interesting correlations between nWASP levels and clinical observations of lung cancer, particularly with phenotypes indicative of invasive and advanced cancers. Survival has been shown to correlate with TNM staging and nodal involvement as expected [32], with prognosis lower in patients with node positive and high TNM stage cancers. nWASP activity has been shown to correlate with patient survival with significantly poorer prognosis for patients who are found to have high levels of nWASP transcript expression. Furthermore, nWASP transcript expression levels are shown to correlate with TNM stage and lymph node involvement highlighting nWASP as a potential biomarker of more aggressive lung cancers phenotypes. These findings suggest that nWASP may be involved in the invasion of cancer cells to the lymph nodes and the development of more advanced lung cancers, and hence has a significant correlation with patient survival.

This study has demonstrated that nWASP levels in tumours may correlate with patient smoking habits. This is an interesting finding given that smoking is recognised as a factor related to lung cancer incidence and development. The results from this study infer that the elevated nWASP activity detected in tumours of patients who are long-term smokers may be related to the development of particular types of cancer that are more prevalent in tobacco users.

nWASP levels are elevated in non-small cell carcinoma lung cancer tissues compared with small-cell carcinomas. As such, A-549 and SK-MES-1 cell lines were selected as models for the adenocarcinoma and squamous carcinoma lung cancer sub types respectively in order to carry out further assays to investigate the role of nWASP in non-small cell lung cancer cell behaviour.

Several in vitro studies were carried out to examine the effect of nWASP inhibition, using the agent wiskostatin, and knockdown, using siRNA, on the behaviour of the A-549 adenocarcinoma cell line and the SK-MES-1 squamous carcinoma cell line. Growth was significantly impaired in both lung cancer cell lines by nWASP inhibitor treatment and knockdown. Furthermore, invasion of SK-MES-1 cell lines is significantly affected by both in vitro invasion assays and through ECIS analysis where the healing of an electrical wound is impaired by nWASP inhibition and knockdown. Wiskostatin appears not to have an effect on the invasion of A-549 cells in in vitro invasion assays but ECIS analysis using A-549 nWASP knockdown cells appears to demonstrate that nWASP activity does affect the invasive/migratory properties of A-549 cells following an electrical wounding. This difference could perhaps be because the inhibitor may not be fully effective on inhibiting nWASP in the cells after the length of the ECIS experiment or perhaps by a change in the attachment behaviour of the A-549 cells, as shown in the Matrigel adhesion assay, which could account for the change in the capacitance. The motility and migratory properties of A-549 cells appear to be significantly impaired by nWASP inhibition. These properties are affected over short time periods of less than 24 h, at which point no significant effect on A-549 growth following wiskostatin treatment is seen, so this effect on cell behaviour is considered to be independent of changes to cell proliferation.

The differences observed in behaviour of these cells lines in response to nWASP inhibition and knockdown could be explained by the origins of the A-549 and SK-MES-1 cell lines. Squamous carcinoma cell types are more invasive and migratory compared with adenocarcinoma cells [25]. It has been shown here that nWASP expression correlates with more advanced lung cancer phenotypes and in particular lymph node metastasis. As such, the effect of nWASP inhibition and knockdown on invasive cell behaviours may be more apparent in the more invasive SK-MES-1 squamous carcinoma cell line.

The experiments demonstrate that nWASP activity has a role in controlling the invasive and migratory behaviour of cancer cells, which is supported by previous studies [16–20], and that the response of the cell lines used in this study to nWASP inhibition is different. A reduction in the adhesive properties of both lung cancer cell types in response to nWASP inhibition was suggested too, although more significantly in A-549 cells. This effect on the adhesive and spreading properties is also highlighted in both cell lines through ECIS.

A possible explanation for the effect on adhesion and other behaviours observed in the A-549 and SK-MES-1 cell lines following nWASP inhibition is provided through immunofluorescence studies. These demonstrate fewer paxillin rich adhesions are found on the periphery of both cell types following nWASP inhibition and knockdown. Paxillin is a key protein which localises to site of focal adhesions between the cell and its surroundings and an increase in paxillin signalling has been linked to enhanced metastatic potential [26–28]. FAK activity, another molecule which forms and integral part of focal adhesions and has also been highlighted as a key molecule in lung cancer invasion and migration [33, 34], is also affected by nWASP activity in A-549 cells. FAK phosphorylation at tyrosine 925, which is decreased in nWASP knockdown A-549 cells, has also been implicated in the control of FAK-mediated cell migration and cell protrusion, further highlighting a role of nWASP in cell migration [35]. The observation of loss of paxillin and FAK activity following nWASP inhibition is therefore indicative of a reduction in invasive potential. Previous reports agree with this conclusion which implicates nWASP in the control of membrane protrusion formation and structure in cancer cells [29–31]. However, direct signalling mechanisms linking nWASP to the control of focal adhesions are yet to be identified in the context of lung cancer invasion.

## Conclusions

The findings presented in this study suggest that nWASP inhibition in lung cancer cell types does in fact alter cell morphology with respect to the level of paxillin-rich membrane structures the cells are able to produce. This is may be due to the role of nWASP in the organisation of the actin cytoskeleton. Since the importance of actin-rich membrane protrusions in cell movement and cancer invasion is well understood, it is clear that nWASP is a key molecule of interest with respect to controlling these cell behaviours. From the results of the functional assays it is even more apparent that nWASP activity is important in the control of cellular movement and, when considered alongside findings showing elevated nWASP levels in metastatic and advanced stage lung cancer tissues, this study concludes that nWASP is a key molecule of interest with respect to lung cancer invasion.

## Abbreviations

DMEM: Dulbecco’s modified Eagle’s medium
ECACC: European Collection of Animal Cell Culture
ECIS: Electric cell-substrate impedance sensing
FCS: Foetal calf serum
QPCR: Quantitative polymerase chain reaction
